# Transcriptome-Based Survival Analysis Identifies *MAP4K4* as a Prognostic Marker in Gastric Cancer with Microsatellite Instability

**DOI:** 10.3390/cancers17030412

**Published:** 2025-01-26

**Authors:** Alvaro De Jesus Huamani Ortiz, Anthony Vladimir Campos Segura, Kevin Jorge Magaño Bocanegra, Mariana Belén Velásquez Sotomayor, Heli Jaime Barrón Pastor, Yesica Llimpe Mitma de Barrón, Ruy Diego Chacón Villanueva, Alexis Germán Murillo Carrasco, César Alexander Ortiz Rojas

**Affiliations:** 1 Molecular Medicine Research and Teaching Group (MEDMOL), Faculty of Medicine, National University of San Marcos, Lima 15081, Peru; alvaro.huamani@unmsm.edu.pe (A.D.J.H.O.); helibarron@unmsm.edu.pe (H.J.B.P.); yllimpem@unmsm.edu.pe (Y.L.M.d.B.); 2 Immunology and Cancer Research Group (IMMUCA), OMICS, Lima 15001, Peru; bladimircs001@gmail.com (A.V.C.S.); marianavelasquez11@gmail.com (M.B.V.S.); ruychaconv@alumni.usp.br (R.D.C.V.); 3 Clinical and Functional Genomics Group, International Center of Research CIPE, A.C. Camargo Cancer Center, Sao Paulo 01509-010, Brazil; 4 Department of Molecular Biomedicine, Center for Research and Advanced Studies (CINVESTAV-IPN), Mexico City 07360, Mexico; kevinmb1999@gmail.com; 5 Faculty of Medicine, Southern Scientific University, Lima 150142, Peru; 6 Department of Pathology, School of Veterinary Medicine, University of São Paulo, São Paulo 05508-900, Brazil; 7 Center for Translational Research in Oncology (LIM/24), Hospital das Clínicas, Faculty of Medicine, University of São Paulo (HCFMUSP), São Paulo 01246-000, Brazil; 8 Comprehensive Center for Precision Oncology, University of São Paulo, São Paulo 01246-000, Brazil; 9 Medical Investigation Laboratory in Pathogenesis and Targeted Therapy in Onco-Immuno-Hematology (LIM/31), Hospital das Clínicas, Faculty of Medicine, University of São Paulo (HCFMUSP), São Paulo 01246-000, Brazil

**Keywords:** stomach neoplasms, gene expression, microsatellite instability, biomarker

## Abstract

**Background/Objectives**: Gastric cancer (GC) is a highly aggressive malignancy with diverse molecular subtypes. While microsatellite instability (MSI) GC generally carries a favorable prognosis, a subset of patients experiences poor outcomes, highlighting the need for refined prognostic markers. **Methods:** This study utilized transcriptomic and clinical data from two independent cohorts, The Cancer Genome Atlas (TCGA) and the Asian Cancer Research Group (ACRG), to identify novel prognostic genes in MSI-GC. Results: Through rigorous survival analysis, we identified high *MAP4K4* expression (*MAP4K4^high^*) as an independent and robust predictor of poor overall survival (OS) and disease-free survival (DFS) specifically within the MSI-GC subtype. *MAP4K4^high^* was associated with increased hazard ratios for both OS and DFS in both cohorts, even after adjusting for clinicopathological factors. Further analysis revealed that *MAP4K4^hig^*^h^ MSI-GC tumors exhibit a distinct molecular profile characterized by increased extracellular matrix remodeling, epithelial–mesenchymal transition, and a microenvironment enriched in monocytes and cancer-associated fibroblasts (CAFs). Notably, a subgroup of MSI-GC patients with a CIN-like phenotype and high *MAP4K4* expression exhibited particularly dismal outcomes. Conclusions: Our findings establish *MAP4K4* as a promising prognostic biomarker for risk stratification in MSI-GC and suggest its potential role in driving aggressive tumor behavior through modulation of the tumor microenvironment.

## Simple Summary

This study identifies the high expression of *MAP4K4* as a biomarker of poor prognosis in microsatellite instability gastric cancer. *MAP4K4*’s prognostic significance was specific to MSI-GC than other molecular GC subtypes. Further analysis revealed that tumors with high *MAP4K4* expression exhibit enhanced extracellular matrix remodeling, epithelial–mesenchymal transition, and distinct immune microenvironment characteristics, such as increased monocyte and CAF infiltration. These findings position *MAP4K4* as a promising marker for risk stratification and a potential therapeutic target in MSI-GC.

## 1. Introduction

GC is characterized by significant heterogeneity, with diverse clinical, histological, and molecular factors influencing disease presentation and patient prognosis. Traditional classification systems were developed to deepen this heterogeneity like Lauren and WHO classifications [1,2,3], based on histology, and the TNM staging system, which are used for diagnosis, treatment planning, and prognostication in GC [4]. In the last decade, molecular techniques have allowed for the refinement of traditional classifications by incorporating genetic and other molecular features. Thus, The Cancer Genome Atlas (TCGA) and the Asian Cancer Research Group (ACRG) studies have proposed molecular subtypes of GC by using genomic and transcriptomics approaches, respectively [5,6]. Both of these molecular classifications include the microsatellite instability (MSI) subtype, characterized by the dysfunction of the mismatch repair (MMR) genes. The hallmark of this dysfunction is the alteration in the length of microsatellite DNA sequences. In GC, patients with MSI (MSI-GC) have favorable prognosis associated with early diagnosis (TNM stages I or II) and lower risk of recurrence and metastasis [7,8]. MSI-GC patients receiving standard treatment exhibit higher survival rates, with over 70% of patients alive and over 80% of patients remaining relapse-free at 5 years of follow-up [9,10,11,12]. Despite these favorable prospects, a subset of MSI-GC patients does not respond to standard treatments, which highlights the complex biology of these tumors [13,14]. Similarly, heterogeneity in immunotherapy response has been observed in these patients [15,16]. In this sense, new biomarkers are necessary to identify patients with low chances of responding to established therapies.

Tumor transcriptomes represent a potential arsenal of new biomarkers for advancing precision medicine for MSI-GC patients. Some efforts have been made to identify gene expression signatures related to prognosis, which may be associated with standard therapy and immunotherapy response [17,18,19]. Unfortunately, the large number of genes comprising these signatures could be a limitation for clinical application [20,21]. In the present study, we aimed to perform a transcriptomic analysis to identify genes whose expression profiles could serve as prognostic markers for MSI-GC, which could then contribute to the development of more personalized treatment strategies for this specific patient population.

## 2. Materials and Methods

### 2.1. Patient Cohorts and Gene Expression Profiling

Publicly available clinical and transcriptomic data of two adult MSI-GC cohorts were included in this study. First, we used data from patients from the stomach cohort of The Cancer Genome Atlas Stomach Adenocarcinoma (TCGA-STAD) project (*n* = 68) [5]. RNA-seq and clinical data were retrieved from the Firebrowse data portal site (http://firebrowse.org/, accessed on 1 April 2024). Next, data from the Asian Cancer Research Group (ACRG) cohort (*n* = 68) were included [6]. These data correspond to a microarray data set (GSE66229) that was retrieved from the Gene Expression Omnibus (GEO) database (https://www.ncbi.nlm.nih.gov/geo/, accessed on 1 April 2024). Transcriptome data of the TCGA cohort were generated using HiSeq 2000 (Illumina, San Diego, CA, USA) and included information on 20,508 genes. Data from ACRG were generated using Human Genome U133 Plus 2.0 (Affymetrix, Santa Clara, CA, USA), which contained probes for 23,520 genes.

### 2.2. Statistical Analysis

Patients were divided into high- and low-expression groups based on the optimal cutoff point determined by receiver operating characteristic (ROC) curve analysis of overall survival (OS) data for each gene. For that, the Youden index [22], which maximizes the balance between sensitivity and specificity, was used. Additionally, in order to address the uncertainty of the optimal cutoff point, we set the bootstrapping parameter to 50. Also, we considered a cutoff value that preserves at least 20% of the total sample size in each group, avoiding potential overfitting, small-sample-size groups, and biased cutoff value selection. Furthermore, if multiple cutoff points were obtained for each gene, we selected the cutoff value closest to the median as recommended previously [23]. Then, statistical tests were used in sequence to filter genes whose gene expression is associated with survival in MSI-GC. First, univariate and multivariate Cox regression for OS and disease-free survival (DFS) were used after the dichotomization process. For multivariate regression, we considered age, sex, Lauren categorical classification, tumor stage (T), nodal status (N), and primary tumor site as possible confounding factors. Afterward, only genes generating a survival AUC > 0.5, sensitivity > 0.5, and specificity > 0.5 were considered as potential biomarkers. Next, we used the R package “survivalpwr” to calculate the Cox regression power for OS and DFS, using a threshold of 80%. Only genes passing the analysis in both TCGA and ACRG were considered as robustly associated with prognosis.

Fisher’s exact test and the Mann–Whitney test were used to study clinical variables. To describe the genes robustly associated with prognosis, survival AUC up to a 3-year follow-up and Kaplan–Meier (KM) plots were generated for each cohort. All calculations were performed using R software v4.4.1 (CRAN Project, www.r-project.org, accessed on 1 April 2024).

### 2.3. Gastric Cancer Subtyping by GCclassifier

To identify GC molecular subtypes, we used the R package GCclassifier [24]. GCclassifier uses gene expression data to provide scores for each molecular subgroup, thus identifying MSI-, EBV-, CIN-, and GS-like phenotypes. This classification was applied only in cases where original molecular classification, established by original studies, was absent. K-means clustering, using the silhouette method, was used to establish molecular groups.

### 2.4. Gene Set Enrichment Analysis

Gene set enrichment analysis (GSEA) using Broad Institute software, version 4.2.3 (http://software.broadinstitute.org/gsea/index.jsp, accessed on 1 May 2024), was performed to find biological processes associated with gene expression [25]. Huma C2-7 gene set collections, including those of Gene Ontology (GO), Kyoto Encyclopedia of Genes and Genomes (KEGG), and Reactome databases, were included in our analysis. Enrichment scores were calculated based on Kolmogorov–Smirnov statistics tested for significance using 1000 permutations. Spearman’s correlation was used as a metric for ranking genes. A pathway was considered enriched when an FDR q-value was <0.05. Then, redundancy analysis was performed by calculating meet–min similarity indexes. With the generated similarity matrix, hierarchical clustering was performed to group similar gene sets.

### 2.5. Prediction of Immune Cell Infiltration

To understand if a specific gene signature is associated with a microenvironment composition that could explain tumor aggressiveness, we predicted tumor microenvironment (TME) cellularity by identifying immune and stromal cell signatures through deconvolution algorithms based on transcriptomic data. Thus, CIBERSORT, a web application from Stanford University that estimates cellular composition abundance in tissues (http://cibersort.stanford.edu, accessed on 1 June 2024) [26], EPIC, an analytical tool that evaluates immune cell proportions such as B lymphocytes, cancer-associated fibroblasts (CAFs), CD4, CD8, macrophages, and natural killer cells (http://epic.gfellerlab.org, accessed on 1 June 2024) [27], xCell, an in silico cellular enrichment simulation containing 64 types of immune and stromal cells in tissues and cells (https://xcell.ucsf.edu/, accessed on 1 June 2024) [28], MCP-counter, consisting of 8 immune cell populations and 2 stromal populations in tissues (http://134.157.229.105:3838/webMCP/, accessed on 1 June 2024) [29], and quanTIseq, predicting the infiltration of 10 immune cell populations in tumor samples (http://icbi.at/quantiseq, accessed on 1 June 2024) [30], were used. All algorithms were obtained from the web tool TIMER 2.0 [31], a comprehensive program for evaluating tumor immune signatures (http://cistrome.org/TIMER, accessed on 1 June 2024).

## 3. Results

### 3.1. MAP4K4 Expression Is a Robust, Independent Prognostic Marker in MSI-GC

**Figure 1:**
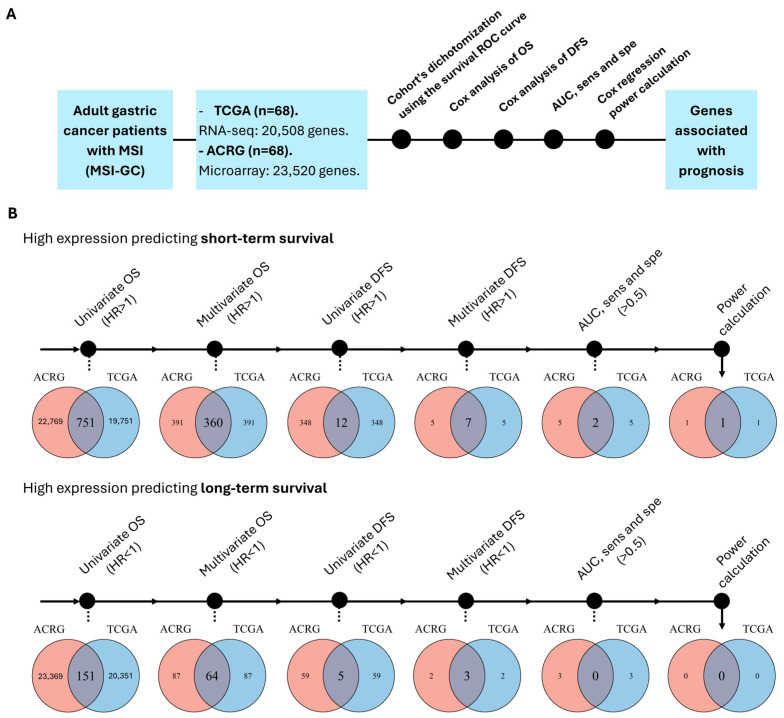
Identifying prognostic gene expression biomarkers in MSI-GC. (**A**) Workflow of the statistical strategy for identifying genes associated with prognosis. (**B**) Venn diagrams showing how many genes were found at each step. The process was split based on whether the high gene expression means shorter or longer survival.

To identify genes related to survival in MSI-GC, a gene-by-gene survival association analysis was performed using the transcriptomic and clinical data of two independent cohorts (TCGA and ACRG; see Section 2). First, we selected GC patients with MSI (determined in the original studies by PCR and IHC [5,6]). Then, each cohort was dichotomized according to the expression levels of each gene of the transcriptome by using the optimal cutoff points (see Section 2). Next, OS and DFS Cox regressions, survival AUC, sensitivity and specificity, and statistical power calculation were used in sequence to filter genes associated with prognosis (Figure 1A). After the evaluation of >20,000 genes in each cohort, we found that the expression of only 1 gene, *MAP4K4*, was associated with prognosis by predicting short-term survival in both cohorts (Figure 1B).

**Figure 2:**
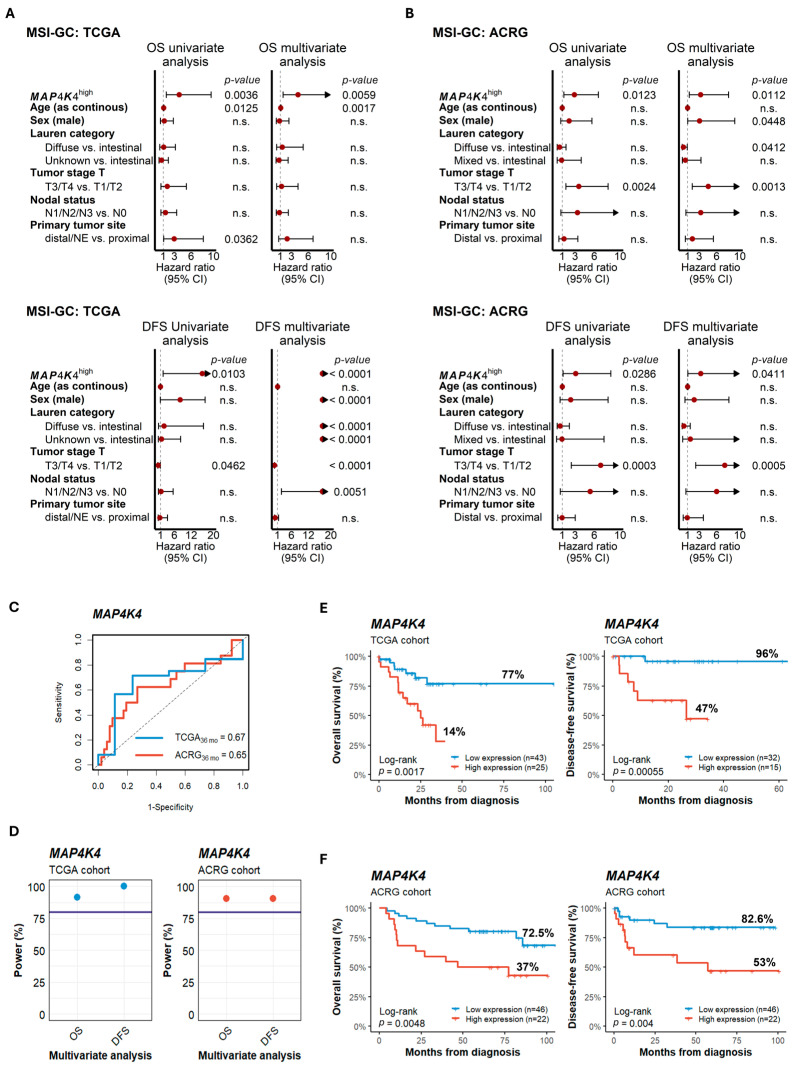
Prognostic evaluation of *MAP4K4* expression in MSI-GC. (**A**,**B**) Univariate and multivariate Cox regression for OS (upper panels) and DFS (bottom panels) evaluated in the TCGA (**A**) and ACRG (**B**) datasets indicating an association of *MAP4K4*^high^ with poor prognosis. (**C**) Survival ROC curves showing the survival prediction capacity of *MAP4K4* up to 3 years of follow-up. (**D**) Power calculation for Cox regression indicating that the sample size was enough to detect an association of *MAP4K4* expression with survival. The horizontal lines indicate 80% as the threshold. (**E**,**F**) KM plots showing that *MAP4K4*^high^ patients have significantly lower survival rates in both the TCGA (**E**) and ACRG (**F**) cohorts. n.s.: not significant (*p* > 0.05).

An elevated expression of *MAP4K4* (hereafter referred to as *MAP4K4*^high^) was significantly associated with poorer survival compared to patients with lower *MAP4K4* expression (*MAP4K4*^low^). In the TCGA dataset, Cox regression analysis indicated hazard ratios (HRs) of 3.9 (*p* = 0.0036) and 16.0 (*p* = 0.0103) for OS and DFS, respectively. After accounting for potential confounding clinical factors, the corresponding HRs were 4.2 (*p* = 0.0059) and >20 (*p* < 0.0001) (Figure 2A). Similarly, for the ACRG cohort, the HRs were 2.9 (*p* = 0.0123) and 3.1 (*p* = 0.0286) in the OS and DFS univariate analysis, where for multivariate analysis the HRs were 3.2 (*p* = 0.0112) and 3.3 (*p* = 0.0411), respectively (Figure 2B). Moreover, the survival AUCs were >0.6, and the statistical power for OS and DFS was >90% in both the TCGA and ACRG cohorts (Figure 2C,D), indicating the reliability of the prognosis predictive capacity of *MAP4K4* expression. Finally, KM plots indicated lower survival rates for *MAP4K4*^high^ patients compared to those with *MAP4K4*^low^ in both TCGA and ACRG. Thus, for TCGA, the OS rates were 14% and 77% (log-rank, *p* = 0.0017), while the DFS rates were 47% and 96% (log-rank, *p* = 0.0006) (Figure 2E), respectively. Similarly, the OS rates for ACRG were 37% and 72.5% (*p* = 0.0048), while for DFS the OS rates were 53% and 82.6% (Figure 2F), respectively.

To confirm if the association of *MAP4K4* expression with prognosis is not explained by other relevant clinical or genetic variables, we compared these characteristics between *MAP4K4*^high^ and *MAP4K4*^low^ MSI-GC patients. Our analysis revealed, in both the TCGA and ACRG cohorts, no significant association between *MAP4K4* expression and clinical characteristics, including age, tumor staging, and Lauren classification (Table 1 and Table 2). These results are in line with our multivariate Cox regression analyses.

### 3.2. MAP4K4 Is a Prognostic Biomarker Only in MSI-GC and Not in Other GC Molecular Subtypes, Identifying a Very Adverse Group in MSI with the CIN-like Phenotype

**Figure 3:**
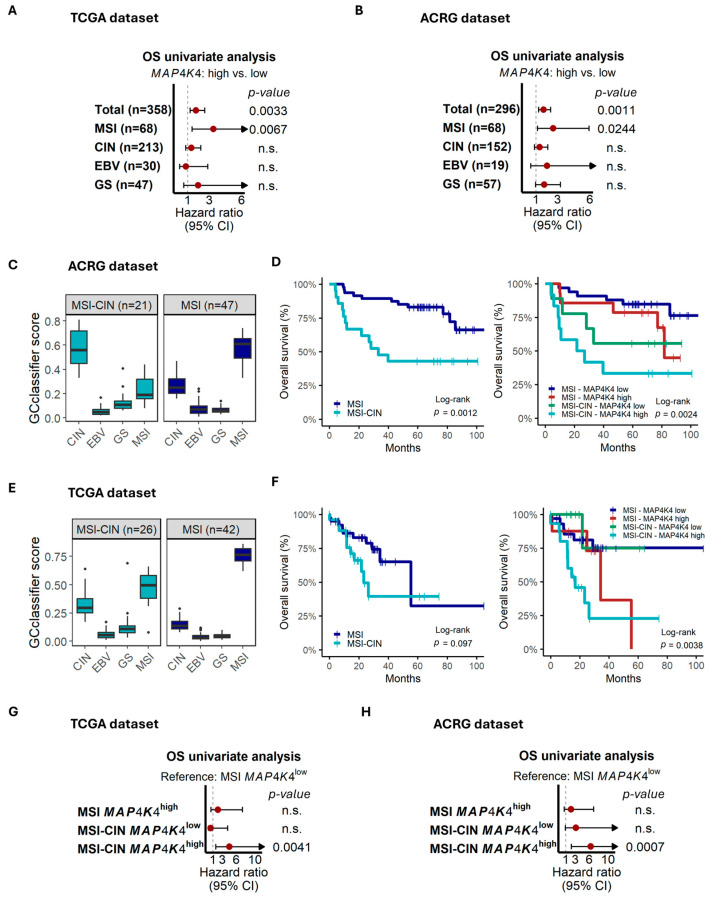
Impact of *MAP4K4* expression on prognosis in GC molecular subtypes. (**A**,**B**) The Cox regression analysis indicates *MAP4K4* as a prognosis biomarker for the MSI subtype but not for other molecular subtypes, in both the TCGA and ACRG datasets. (**C**) In ACRG, the CIN-like phenotype was recognized in some MSI-GC patients, named MSI-CIN. (**D**) MSI-CIN had lower survival rates in comparison with non-CIN MSI cases. (**E**) Interestingly, *MAP4K4*^high^ in MSI-CIN defines a very poor prognosis group in MSI-GC. (**E**,**F**) These results were validated in the TCGA dataset. (**G**,**H**) Finally, Cox regression confirms MSI-CIN *MAP4K4*^high^ as a very aggressive group. n.s.: not significant (*p* > 0.05).

Recently, Zhang et al. have reported *MAP4K4* expression as a prognostic marker in GC [32], indicating possible roles of *MAP4K4* in other molecular GC subtypes. Therefore, we evaluated the survival association of *MAP4K4* expression in chromosomal instability (CIN), Epstein–Barr virus (EBV), and genome-stable (GS) molecular subtypes established by the TCGA study [5]. As shown in Figure 3A, *MAP4K4* predicted prognosis only in the MSI subgroup. To validate this result, we applied the *GCclassifier* algorithm to establish these molecular subgroups in the ACRG dataset (see Section 2). Again, *MAP4K4* was prognostically associated only in the MSI group (Figure 3B). Interestingly, when it was time to apply *GCclassifier* to the ACRG dataset, we observed that some MSI-GC patients had higher CIN scores, representing a CIN-like phenotype within the MSI molecular subtype (Supplementary Figure S1A). After applying clustering methods to MSI-GC patients based on *GCclassifier* scores, we found two clusters, one of them characterized by higher CIN scores, hereafter referred to as MSI-CIN (Figure 3C and Figure S1B). MSI-CIN patients showed lower survival in comparison with the non-CIN MSI cases but at the same time identified a very poor prognosis group when combined with *MAP4K4* expression (Figure 3D). To verify these findings, we applied *GCclassifier* to the TCGA MSI-GC patients. Again, a cluster of MSI-GC patients with high CIN scores was identified (Figure 3E and Figure S1C,D). Although the MSI-CIN cluster was not associated with lower survival, we confirm that MSI-CIN patients with a high expression of *MAP4K4* represent a subgroup with very adverse risk (Figure 3F). Finally, Cox regression confirmed that the MSI-CIN *MAP4K4*^high^ tumors are highly aggressive (Figure 3G,H). Further research needs to be conducted to underscore the biology behind this phenotype, which represents very unfavorable risk.

### 3.3. MAP4K4^high^ MSI-GC Tumors Exhibit Increased Extracellular Matrix Remodeling Activity, Epithelial–Mesenchymal Transition (EMT), and a Distinct Microenvironment Composition

**Figure 4:**
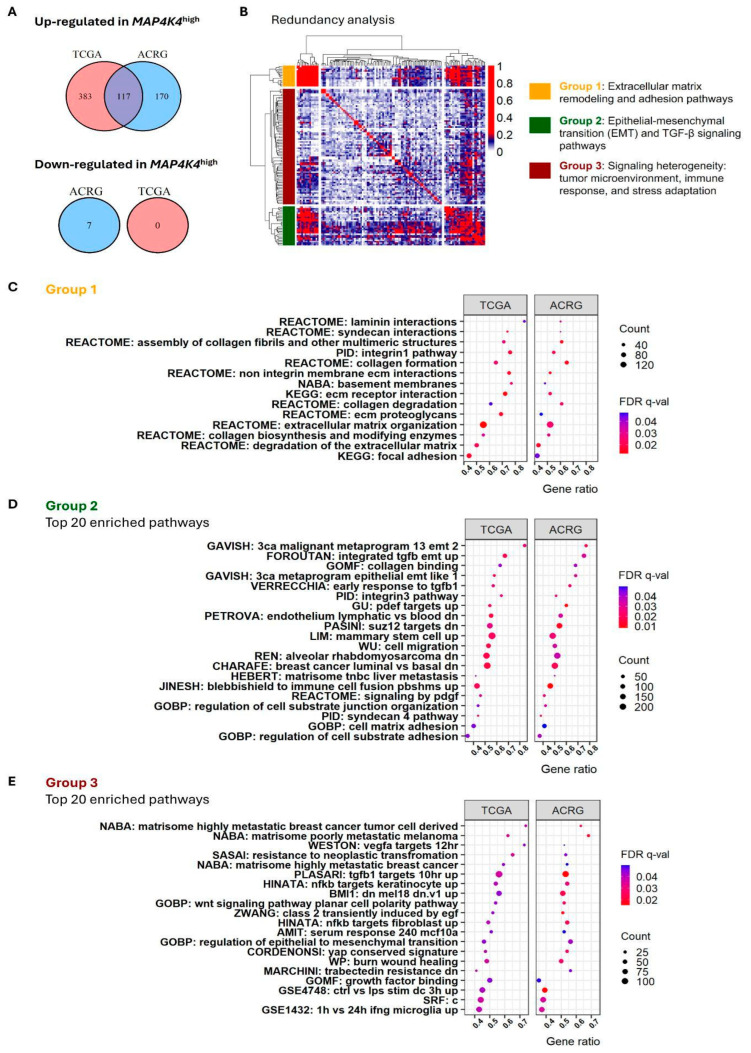
The gene set enrichment analysis revealed that *MAP4K4* expression is associated with pathways involved in aggressive tumor behavior. (**A**) The enrichment analysis identified 117 upregulated pathways in *MAP4K4*^high^ tumors. (**B**) The redundancy analysis revealed three distinct groups of biological pathways. (**C**–**E**) The first group comprised pathways related to extracellular matrix (ECM) remodeling (**C**), the second consisted of pathways associated with epithelial–mesenchymal transition (EMT) (**D**), and the third, a heterogeneous group, included pathways involved in cellular signaling, immune response, and other processes (**E**).

**Figure 5:**
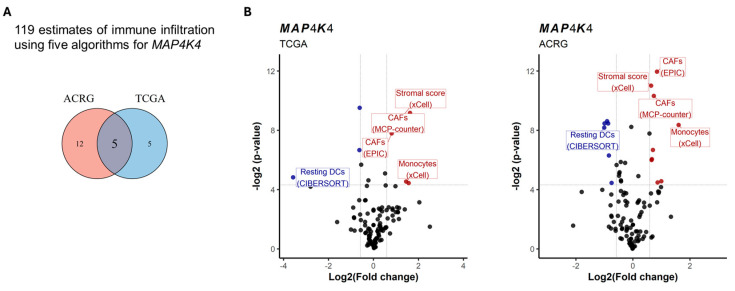
*MAP4K4*^high^ MSI-GC tumor microenvironments are enriched with CAFs and monocytes. Deconvolution analysis using the TIMER 2.0 database was performed. From 119 estimates, just 5 were common between TCGA and ACRG cohorts (**A**), these being CAFs and monocytes differentially infiltrated in tumors with *MAP4K4*^high^ (**B**).

To delve into the molecular mechanism that could explain the outcomes of *MAP4K4*^high^ MSI-GC patients, we performed a biological pathway enrichment analysis using gene set collections from GSEA (see Section 2). After the evaluation of C2-7 human collections, which include Gene Ontology (GO) gene sets, a total of 500 and 287 pathways were enriched in *MAP4K4*^high^ tumors of the TCGA and ACRG datasets, respectively (Supplementary Tables S1 and S2). We selected 117 common enriched pathways between both datasets (Figure 4A) and performed a redundancy analysis (see Section 2). The redundancy analysis resulted in three groups of pathways: one related to extracellular matrix remodeling, another associated with epithelial–mesenchymal transition (EMT), and the last involved in cellular signaling (Figure 4B). Group 1 corresponded to 14 pathways involved in the structural and functional organization of the extracellular matrix, cell–matrix interactions, and integrin-mediated adhesion, underscoring *MAP4K4*’s role in promoting aggressive tumor traits in MSI-GC (Figure 4C). Group 2 included processes that govern EMT, cell migration, and adhesion reorganization, indicating that *MAP4K4* could facilitate EMT and TGF-β-driven metastatic processes. Otherwise, group 3 was heterogeneous, including gene sets with low or any redundancy. The pathways in this group reflect the influence of *MAP4K4* on the tumor microenvironment, immune interactions, and cellular responses to stress, suggesting multifaceted roles in MSI-GC progression. Finally, we performed a transcriptome deconvolution analysis to evaluate the microenvironment composition and immune cell infiltration into the *MAP4K4*^high^ MSI-GC tumors, for which five algorithms corresponding to 119 estimates of immune infiltration were used (see Section 2). In both the TCGA and ACRG datasets, five signatures were found associated with *MAP4K4*^high^ tumors. From these, four signatures were related to monocytes and CAFs, while resting DCs were under-represented in patients with *MAP4K4*^low^ (Figure 5A,B).

## 4. Discussion

Most patients with microsatellite instability gastric cancer (MSI-GC) have a favorable prognosis and respond well to standard treatments; however, a subset does not benefit from these therapies [10,13,14]. Furthermore, although classic classification systems provide prognostic assessments and guide therapeutic strategies in gastric cancer, they do not adequately consider the molecular heterogeneity of MSI tumors [14]. This highlights the need to identify more specific biomarkers to better understand the prognosis and biological complexity of MSI. Therefore, we performed a transcriptome-based survival analysis in two independent cohorts to identify genes associated with prognosis in MSI-GC. By considering only genes whose expression profile can predict prognosis in both cohorts, we found that high *MAP4K4* expression is an independent predictor of lower overall survival (OS) and disease-free survival (DFS) rates in MSI-GC. *MAP4K4* (also known as HGK or NIK) is a serine/threonine kinase belonging to the Ste20 family of protein kinases, which plays a significant role in immunity, inflammation, metabolic disorders, cardiovascular diseases, and cancer [33]. Previous studies have observed that *MAP4K4* is associated with poorer prognosis, accelerated progression, higher recurrence rates, and an increased number of metastatic lymph nodes in various cancers, including pancreatic ductal adenocarcinoma, colorectal cancer, prostate cancer, lung adenocarcinoma, acute myeloid leukemia, and hepatocellular carcinoma [34,35,36,37,38,39,40]. Interestingly, *MAP4K4* expression has been previously studied in gastric cancer, demonstrating its prognostic predictive ability [32]. However, our study suggests that the prognostic value of *MAP4K4* may not be applicable to all molecular subtypes of gastric cancer, with MSI-GC tumors being a suitable group for this biomarker. In addition to its association with prognosis, *MAP4K4* expression has been linked to other clinical features in gastric cancer. For example, Tong et al. [41] reported an association between *MAP4K4*^high^ and more advanced tumor stages, although this finding was not confirmed by other studies [32]. Similarly, our study found that *MAP4K4* expression is not associated with pathological stages. Furthermore, no other clinical variable was associated with *MAP4K4* expression in MSI-GC, confirming the role of this gene as an independent prognostic marker.

To understand why *MAP4K4* expression is associated with MSI-GC aggressiveness, we compared biological pathway signatures between *MAP4K4*^high^ and *MAP4K4*^low^ tumors, finding that extracellular matrix (ECM) remodeling, epithelial–mesenchymal transition, cellular signaling, immune response, and other processes were upregulated in *MAP4K4*^high^ tumors. The role of *MAP4K4* in ECM remodeling has recently been established. Alberici Delsin et al. [42] have shown that *MAP4K4* promotes focal adhesion disassembly, which is essential for cell detachment and movement. By modulating focal adhesion dynamics, *MAP4K4* can promote cancer cell invasion and metastasis by enabling cells to detach from the primary tumor and migrate to distant sites. In this context, EMT—a known process where epithelial cells lose their cell–cell adhesion and polarity, acquiring mesenchymal characteristics—can be promoted by EMT-related transcription factors modulated by *MAP4K4*, contributing to the acquisition of mesenchymal properties [42]. Similarly, we observed the enrichment of TGF-beta pathways in *MAP4K4*^high^ tumors. Interestingly, we did not observe higher frequencies of metastasis cases in *MAP4K4*^high^ patients. However, the clinical data presented here correspond to diagnostic data; it is possible that metastasis events are more frequent in *MAP4K4*^high^ patients at the time of relapse. In addition, it has been demonstrated that *MAP4K4* influences the organization and dynamics of the actin cytoskeleton, which is essential for cell motility and which is in line with our enrichment analysis [42]. Also, we found *MAP4K4* expression to be involved in response to various stimuli, including growth factors and cytokines. These stimuli can activate signaling pathways that regulate cell proliferation, differentiation, and survival [43]. Thus, it has been demonstrated that MAP4K4 can activate JNK and other downstream effectors, modulating cellular responses to various stimuli [44,45]. By modulating these and other signaling pathways, *MAP4K4* can contribute to aberrant cell growth and survival, promoting cancer progression.

By using deconvolution techniques, we found high monocyte infiltration into *MAP4K4*^high^ MSI-GC tumors. Monocyte infiltration is remarkable since these cells could have either antitumoral or protumoral activity [46,47,48]. In the context of tumors, monocytes can differentiate into several types of tumor-associated macrophages (TAMs), some of them, like M2 macrophages, favoring tumor progression by promoting immunosuppression and enhancing metastasis [49]. However, the possible modulation of monocytes and macrophages exerted by *MAP4K4* in the tumor microenvironment (TME) needs further research. We also found signatures of high cancer-associated fibroblast (CAF) abundance in *MAP4K4*^high^ tumors. CAFs have been recognized as significant elements within the TME due to their diverse roles in tumor progression and the reduction in survival in GC patients [50,51]. These cells modify their environment by secreting proteins such as cytokines and upregulating genes that promote an infiltrative phenotype and metastasis, resulting in worse progression [52,53,54,55]. Indeed, the presence of CAFs in MSI tumors has been already reported [19]. Mak et al. [56] revealed a connection between low CAF abundance and MSI tumors, while high CAF abundance was associated with MSS status. However, our study suggests that a subgroup of MSI-GC patients—those with high *MAP4K4* expression—could accumulate CAFs. Interestingly, Xu et al. [57] recently reported, after evaluating cell–cell interactions via single-cell technology, that monocytes are poorly differentiated in gastric tumor microenvironments, which can explain why we did not observe M1 or M2 signatures associated with *MAP4K4*. Also, they proposed that tumor progression can be mediated by these infiltrated monocytes after interactions with tumor stromal cells like CAFs.

Resting dendritic cells (DCs), also known as immature DCs, act as a reservoir of potential immune activators, continuously monitoring the environment for antigens [58]. Our deconvolution analysis showed that *MAP4K4*^high^ tumors have a lower abundance of resting DCs. The reason for this association needs to be further explored. However, in line with our results, Hanona et al. [58] showed that, in hepatocellular carcinoma, a higher number of resting dendritic cells in the tumor microenvironment represents better survival rates for patients.

Our results also highlight the potential of the pharmacological inhibition of *MAP4K4*. In this regard, the evidence for the anti-neoplastic role of *MAP4K4* inhibition is still in its early stages but promising. In a murine model of pancreatic cancer, the pharmacological inhibition of *MAP4K4* with GNE-495 suppressed pancreatic cell growth and tumor migration [59]. Also, several models of *MAP4K4* inhibition indicate it as a sensitizer to chemotherapy [60,61,62]. Despite these promising results, preclinical studies have noted potential adverse effects, including weight loss, increased body temperature, and tachycardia [63], highlighting the need for further research to address these concerns before clinical application.

## 5. Conclusions

In summary, our transcriptome-based survival analysis identified the expression profile of *MAP4K4* as a significant independent predictor of prognosis for MSI-GC, being associated with TME composition and modulation of known cancer-associated pathways, like EMT, which highlights its potential as a therapeutic target. While further research is needed to validate these results in independent larger cohorts, this study refines our understanding of the role of *MAP4K4* expression in MSI-GC prognosis.

## Figures and Tables

**Table 1:** Clinical characteristics according to *MAP4K4* expression in MSI-GC patients (TCGA cohort).

Level		Total	*MAP4K4* ^high^	*MAP4K4* ^low^	*p*-Value
*n*		68	43	25	
Age, median [IQR]		70.00 [64.00, 75.25]	70.00 [64.00, 75.00]	70.00 [65.00, 76.00]	0.949
Sex, *n* (%)	Female	35 (51.5)	25 (58.1)	10 (40.0)	0.209
	Male	33 (48.5)	18 (41.9)	15 (60.0)	
Mutation count, median [IQR]		1090.00 [739.25, 1329.25]	1158.00 [749.50, 1355.00]	1043.00 [695.00, 1278.00]	0.321
Histologic grade, *n* (%)	G1	1 (1.5)	0 (0.0)	1 (4.0)	0.323
	G2	19 (27.9)	12 (27.9)	7 (28.0)	
	G3	47 (69.1)	31 (72.1)	16 (64.0)	
	GX	1 (1.5)	0 (0.0)	1 (4.0)	
Tumor stage T, *n* (%)	T1/T2	19 (27.9)	13 (30.2)	6 (24.0)	0.780
	T3/T4	49 (72.1)	30 (69.8)	19 (76.0)	
Nodal status, *n* (%)	N0	30 (44.1)	22 (51.2)	8 (32.0)	0.139
	N1/N2/N3	38 (55.9)	21 (48.8)	17 (68.0)	
Metastasis, *n* (%)	M0	63 (92.6)	41 (95.3)	22 (88.0)	0.609
	M1	3 (4.4)	1 (2.3)	2 (8.0)	
	Unknown	2 (2.9)	1 (2.3)	1 (4.0)	
AJCC TNM staging, *n* (%)	IA	1 (1.5)	1 (2.3)	0 (0.0)	0.411
	IB	1 (1.5)	1 (2.3)	0 (0.0)	
	II	15 (22.1)	12 (27.9)	3 (12.0)	
	IIIA	36 (52.9)	22 (51.2)	14 (56.0)	
	IIIB	2 (2.9)	1 (2.3)	1 (4.0)	
	IV	13 (19.1)	6 (14.0)	7 (28.0)	
Primary tumor site, *n* (%)	Proximal	30 (44.1)	21 (48.8)	9 (36.0)	0.325
	Distal	38 (55.9)	22 (51.2)	16 (64.0)	
Lauren classification, *n* (%)	Intestinal	27 (39.7)	16 (37.2)	11 (44.0)	0.681
	Diffuse	14 (20.6)	8 (18.6)	6 (24.0)	
	Unknown	27 (39.7)	19 (44.2)	8 (32.0)	

IQR: Interquartile range.

**Table 2:** Clinical characteristics according to *MAP4K4* expression in MSI-GC patients (ACRG cohort).

Level		Total	Low	High	*p*-Value
*n*		68	46	22	
Age, median [IQR]		66.00 [60.00, 72.00]	66.00 [60.25, 73.75]	65.00 [60.25, 68.75]	0.405
Sex, *n* (%)	Female	23 (33.8)	16 (34.8)	7 (31.8)	1
	Male	45 (66.2)	30 (65.2)	15 (68.2)	
Tumor stage T, *n* (%)	T1/T2	47 (69.1)	33 (71.7)	14 (63.6)	0.579
	T3/T4	21 (30.9)	13 (28.3)	8 (36.4)	
Nodal status, *n* (%)	N0	16 (23.5)	12 (26.1)	4 (18.2)	0.554
	N1/N2/N3	52 (76.5)	34 (73.9)	18 (81.8)	
Metastasis, *n* (%)	M0	67 (98.5)	46 (100.0)	21 (95.5)	0.324
	M1	1 (1.5)	0 (0.0)	1 (4.5)	
AJCC TNM staging, *n* (%)	IB	14 (20.6)	11 (23.9)	3 (12.0)	0.056
	II	26 (38.2)	19 (41.3)	7 (32.0)	
	IIIA	10 (14.7)	7 (15.2)	3 (20.0)	
	IIIB	9 (13.2)	7 (15.2)	2 (8.0)	
	IV	9 (13.2)	2 (4.3)	7 (28.0)	
Primary tumor site, *n* (%)	Proximal	17 (25.0)	11 (23.9)	6 (16.0)	0.772
	Distal	51 (75.0)	35 (76.1)	16 (8.0)	
Lauren classification, *n* (%)	Intestinal	42 (61.8)	26 (56.5)	16 (64.0)	0.212
	Diffuse	20 (29.4)	14 (30.4)	6 (36.0)	
	Mixed	6 (8.8)	6 (13.0)	0 (0.0)	

IQR: Interquartile range.

## Data Availability

The expression profiles and clinical data in this study are publicly available. The Cancer Genome Atlas Stomach Adenocarcinoma data were obtained from http://firebrowse.org/ (accessed on 1 April 2024), and the data from Asian Cancer Research Group (ACRG) were obtained from https://www.ncbi.nlm.nih.gov/geo/ (accessed on 1 April 2024) with GEO accession GSE66229.

## Supplementary Materials

The following supporting information can be downloaded at https://www.mdpi.com/article/10.3390/cancers17030412/s1, Table S1: Gene Ontology pathways enriched in the TCGA cohort; Table S2: Gene Ontology pathways enriched in the ACRG cohort. Figure S1: *GCclassifier* molecular subtyping and clustering evaluation in MSI-GC.
