# Supplementary figures and images for: Transcriptome-Based Survival Analysis Identifies MAP4K4 as a Prognostic Marker in Gastric Cancer with Microsatellite Instability
Source: Cancers (Basel). 2025 Jan 26;17(3):412. doi: 10.3390/cancers17030412 (PMC11816344; doi:10.3390/cancers17030412)

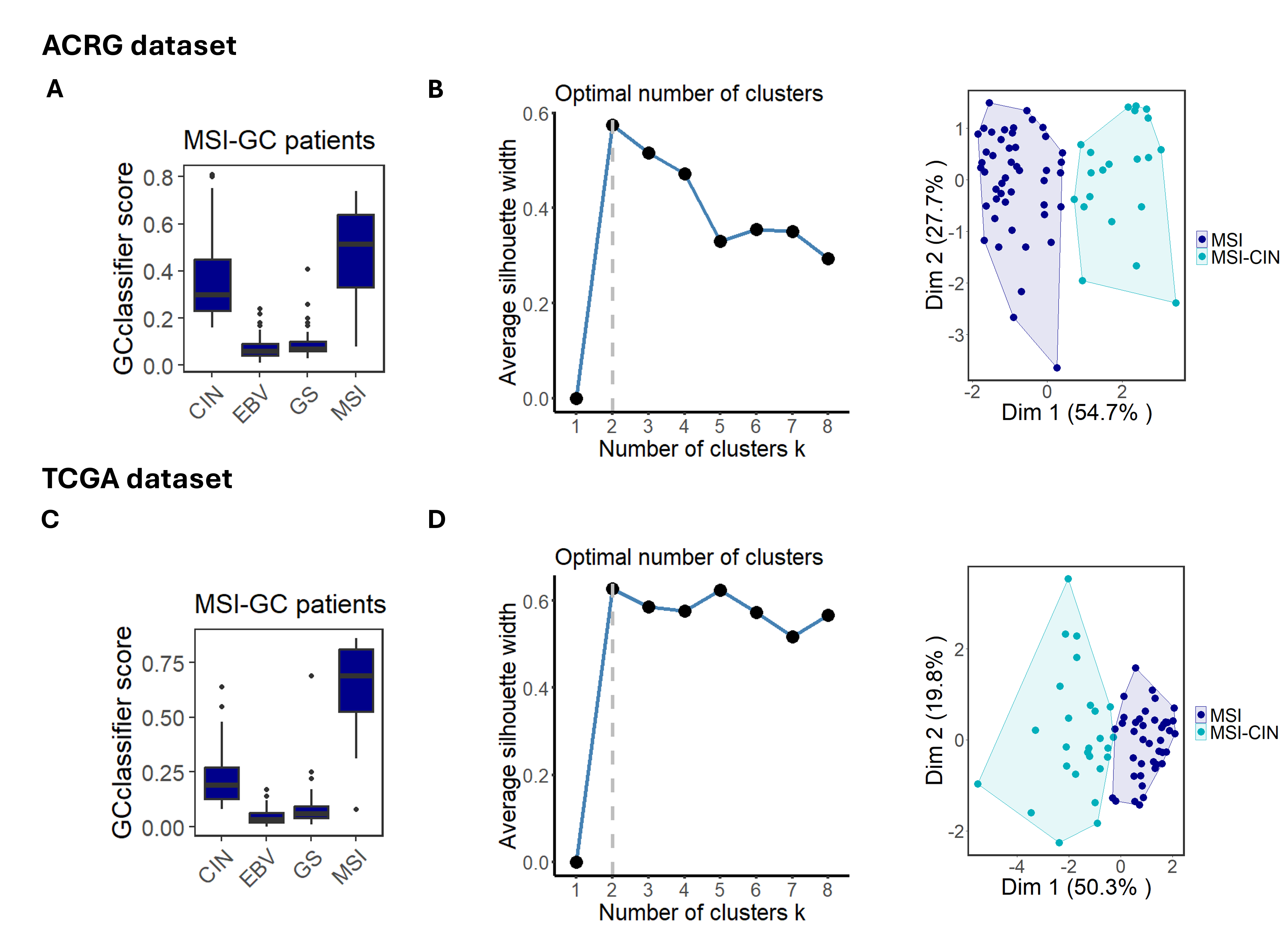

Supplement: Supplementary file 1 [file cancers-17-00412-s001.zip › Supplementary Figure S1.tif]
